# Hydrogen Atom–Coupled Electron Transfer-Driven Anionic Polymerization under Aerobic Conditions

**DOI:** 10.21203/rs.3.rs-10220922/v1

**Published:** 2026-07-17

**Authors:** Saerona Kim, Yao Chung Chang, Muhammed Shameem Kakkattuparambil, Dariya Getya, Udaya Sree Dakarapu, Chang Geun Yoo, Ivan Gitsov, Kwangho Nam, Junha Jeon, Gyu Leem

**Affiliations:** 1 Department of Chemistry and Biochemistry, The University of Texas at Arlington, Arlington, Texas 76019, USA; 2 Department of Chemistry, State University of New York College of Environmental Science and Forestry, Syracuse, New York 13210, USA; 3 Department of Chemical Engineering, State University of New York Environmental Science and Forestry, Syracuse, New York 13210, USA; 4 The Michael M. Szwarc Polymer Research Institute, Syracuse, NY 13210, USA; 5 Department of Biomedical and Chemical Engineering, Syracuse University, Syracuse, New York 13210, USA; 6 Department of Mechanical Engineering, The University of Texas Rio Grande Valley, Edinburg, Texas 78539, USA

## Abstract

Radical and anionic polymerizations are two major routes to synthetic macromolecules, yet they have remained largely separate due to their distinct underlying mechanisms. To bridge these traditionally exclusive methods, we report a strategy that combines the complementary strengths of both methods, fueled by Earth-abundant alkali metal Lewis base and hydrosilanes. Central to this approach is a hydrogen atom-coupled electron transfer (HCET) process that transforms radical-driven initiation into anionic propagation. This method enables the synthesis of a broad spectrum of polymers, demonstrated here by polystyrene homopolymers and poly(styrene)-*block*-poly(methyl methacrylate) copolymers, under user-friendly conditions that tolerate air and use unpurified commodity chemicals. The operational simplicity, oxygen tolerance, and scalability of this method hold promise for sustainable and industrially relevant polymer synthesis.

Polymers are indispensable to modern materials, with the diversity of their structures and properties largely dictated by the methods used to synthesize them. Among the chain-growth methodologies, radical and anionic polymerizations stand out for their versatility in constructing well-defined macromolecular architectures for a wide range of applications in materials science, biotechnology, and advanced coatings^1–3^. Radical polymerization proceeds through reactive, open-shell radical species (**m•**) (Fig. 1a)^4,5^. Because of its operational simplicity, broad monomer scope, and functional group tolerance, it remains one of the most widely used polymerization methods. Development of well-curated combinations of chain transfer agents or transition metal complexes with initiators has imparted living characteristics to these systems, allowing reinitiation of chain growth with minimal termination^6–9^. Despite these advances, industrial implementation remains limited by oxygen and moisture sensitivity, chain transfer or termination processes, and the toxicity of metal catalysts^10,11^.

In contrast, anionic polymerization is initiated by strong nucleophiles and proceeds through reactive, closed-shell anionic species (**m**^−^) generated via two-electron processes (Fig. 1b)^3,12,13^. In the absence of impurities (e.g., water, CO_2_), it is inherently living, thus allowing continuous chain growth and precise control over chain-end characteristics. As a result, this method offers excellent control over molecular weight (M_n_) and dispersity (*Đ*), making it well-suited for the design and synthesis of complex polymer architectures^14^. Nevertheless, its scope and functional group tolerance remain limited by the strong basic conditions required, together with the need for rigorous exclusion of moisture and oxygen and high-purity reagents^15^.

Leveraging these complementary attributes, this study introduces a polymerization strategy that exploits the unique reactivity of hydrosilanes to generate reactive monomeric species. Specifically, reversible Lewis base (LB) activation of hydrosilanes modulates the electronic properties of both the silicon center and the Si–H bond^16–18^. Within the transient, five-coordinate closed-shell silicon anions formed in situ, the hydrogen substituent exhibits a unique *hydrogen-hydride transfer duality*, characterized by its capacity to engage in either hydrogen atom transfer (HAT) or hydride transfer pathways^19–23^. While this mechanistic flexibility offers a powerful bridge between radical and anionic platforms, its strategic application to macromolecular synthesis, together with a fundamental mechanistic understanding of its behavior in polymer systems, remain underexplored, despite its established utility in small molecular synthesis^24–26^.

Motivated by this dual reactivity, we developed a hydrogen atom–coupled electron transfer (HCET)-driven anionic polymerization (HAP) of styrene, mediated by Earth-abundant alkali metal hypercoordinate silicon species (**M**^+^**Si**^−^) under aerobic and user-friendly conditions (Fig. 1c). In HAP, polymerization progresses through two key events: (1) HAT-driven initiation that generates a carbon-centered radical on styrene, followed by (2) single electron transfer (SET) that transforms this radical into an anionic species capable of chain propagation. By directly coupling HAT-driven radical initiation with SET-mediated anionic propagation within a single reaction manifold, this HCET strategy unifies the robustness of radical chemistry with the precision of anionic polymerization.

Mechanistically, the HAT-driven initiation occurs between the hydrogen atom donor (**M**^+^**Si**^−^) and the monomer (**m**) as the acceptor (Fig. 1c). A key requirement in this process is the noncovalent M^+^–π complexation that pre-organizes the reactants and stabilizes a persistent ternary complex comprising nascent carbon-centered radical (**m**^**•**^) within an intimate radical-radical anion pair (**1**). Anionic chain propagation is then realized through a subsequent SET event that generates a carbanion (**m**^−^), where the alkali metal cation serves as an inner-sphere electron-transfer conduit mediating sequential electron flow, depicted in **2**. In detail, this electron transfer from a putative four-coordinate, open-shell bisphenoidal silicon radical anion to a crown ether-bound alkali metal cation is driven by electrostatic interactions and a favorable reduction potential gradient (Supplementary Table 1). The resulting alkali metal radical relays this electron density to the carbon-centered radical, generating an ion-paired carbanion (**m**^−^**M**^+^) that initiates anionic propagation. Importantly, because the intricately interwoven HAT and SET steps operate under intrinsically reductive conditions that outcompete radical oxygen scavenging with minimal chain termination, the system achieves efficient polymerization even in the presence of oxygen. This robust chain-growth behavior permits sequential monomer addition for block copolymerization and exhibits minimal electronic or steric bias toward monomer structure, successfully polymerizing traditionally challenging, electron-rich styrene monomers (e.g., *p*-methoxystyrene)^27,28^.

## Alkali metal ion- and silane-dependent hydrogen atom-coupled electron transfer polymerization (HAP)

**Fig. 2.**
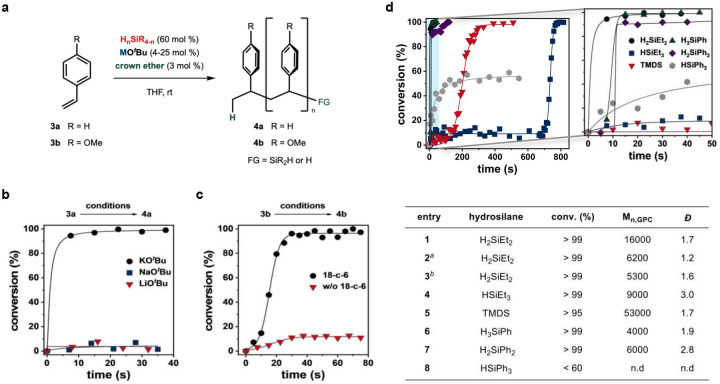
Alkali metal ion- and silane-dependent HCET polymerization. **a**, General polymerization conditions. **b**, Alkali metal ion-dependent HAT initiation [**m** = **3a**, MO^*t*^Bu (M = K^+^, Na^+^, Li^+^), and crown ether]. [m]_o_:[H_2_SiEt_2_]_o_:[MO^*t*^Bu]_o_:[crown ether]_o_ = 267:167:67:7 mM in THF at 25 °C. **c**, Effect of crown ether on polymerization efficiency. Reaction conditions: [**3b**]_o_:[H_2_SiEt]_o_:[KO^*t*^Bu]_o_:[**18-c-6**]_o_ = 267:167:67:7 mM in THF at 25 °C. **d**, Influence of the structure of hydrosilanes on polymer molecular weight and dispersity (**m** = **3a**, KO^*t*^Bu, and H_n_SiR_4-n_). The inset displays an expanded time range (0 to 50 s). Reaction conditions: [**3a**]_o_:[hydrosilane]_o_:[KO^*t*^Bu]_o_:[**18-c-6**]_o_ = 267:167:67:7 mM in THF at 25 °C. GPC analysis summarizes polymers obtained with various hydrosilanes. ^*a*^M_n_ measured in xylene; ^*b*^reaction performed under a pure O_2_ atmosphere. **18-c-6**, 1,4,7,10,13,16-hexaoxacyclooctadecane; TMDS, 1,1,3,3-tetramethyldisiloxane.

In light of our earlier findings on Lewis-base-catalyzed branch-selective HAT olefin hydrosilylation^20^, we investigated the key parameters governing HAP using styrene **3a** as a model monomer under aerobic conditions (Fig. 2a and Supplementary Table 2). We observed a pronounced dependence of HAP on the identity of the alkali metal ion, paired with a bulky *tert*-butoxide Lewis base and a crown ether, as well as on the nature of the hydrosilane. Potassiated *tert*-butoxide (KO^*t*^Bu) proved to be an ideal Lewis base, rapidly promoting polymerization with >99% conversion, whereas smaller cations such as Li^+^ and Na^+^ resulted in poor conversions (<15% and <10%, respectively; Fig. 2b). These ion dependences indicate that the size and coordination behavior of the alkali metal cations affect the formation of a productive ion-pair complex between the vinyl monomer and the five-coordinate, closed shell alkali metal silicon anion^17,22^.

Under identical conditions without crown ether, electron-rich *p*-methoxystyrene **3b** failed to polymerize efficiently (<15% conversion), consistent with the literature precedents^29^. Notably, adding 18-crown-6 ether (**18-c-6**), together with a reduced amount of KO^*t*^Bu yielded successful polymerization to >95% conversion (Fig. 2c). These results suggest that alkali metal cation-coordination by crown ethers plays a critical role in activating electron-rich monomer^19,30,31^. This effect is attributed to effective potassium coordination by **18-c-6**, which lowers the lowest unoccupied molecular orbital (LUMO) of styrene through noncovalent M^+^–π(arene) interactions and generates more weakly coordinated (‘naked’) anions for propagation (Supplementary Table 3)^32^.

Lastly, the influence of hydrosilane structure on HAP reactivity was examined. Steric and electronic factors in hydrosilanes strongly influenced polymerization kinetics, consistent with their role in Lewis base activation of the Si–H bond during HAT (Fig. 2d). In the conversion plots for styrene HAP, dialkyldihydrosilanes (e.g., H_2_SiEt_2_) exhibited rapid polymerization kinetics (Fig. 2d, top), producing polystyrene (PS) with M_n_ = 16,000 (*Đ* = 1.7) in THF and M_n_ = 7,000 (*Đ* = 1.7) in xylene (entries 1 and 2). In contrast, bulkier trialkyl derivative HSiEt_3_ (entry 4) and 1,1,3,3-tetramethyldisiloxane (TMDS, entry 5) displayed marked induction periods at the onset of polymerization, consistent with the previous observation by Grubbs and others^19,33–35^. This behavior likely reflects slower reversible Lewis base activation of bulky HSiEt_3_ or in-situ reorganization of TMDS to H_2_SiMe_2_. Despite these induction periods, both systems achieved >95% conversion; TMDS yielding a high molecular weight polymer (M_n_ = 53,000, *Đ* = 1.7), whereas HSiEt_3_ afforded a low molecular weight with broad dispersity (entry 4). Phenyl-substituted hydrosilanes (entries 6–8) also achieved high conversions–except for HSiPh_3_ (entry 8)–but produced low molecular weight PS due to steric congestion, electronic delocalization, and unproductive silane disproportionation impeding chain propagation.

Remarkably, HAP of unpurified styrene containing inhibitors had virtually no impact on the molecular weight or dispersity (Supplementary Fig. 1), indicating that the basic reaction conditions deactivate inhibitors via deprotonation. Moreover, this HAP protocol remained operative under a pure O_2_ atmosphere (entry 3 and Supplementary Fig. 2), consistent with effective conversion of transient radicals into anionic propagating species. Another notable advantage of this system is its compatibility with unpurified commodity reagents (e.g., monomers, solvent, and related commodity reagents). Under basic conditions, residual water is scavenged by hydrosilanes through hydrolysis, generating silanol intermediates that readily undergo condensation to afford siloxane oligomers. The strong thermodynamic driving force of Si–O bond formation enables efficient consumption of residual moisture, thereby suppressing premature protonation or termination. As a result, uninterrupted anionic propagation can be maintained even in the presence of adventitious water. Collectively, these results demonstrate that HAP is operationally simple and tunable; i.e., alkali metal coordination environment and hydrosilane structure enables control over both molecular weight and dispersity.

## Monomer scope and polymer architecture expansion

**Fig. 3.**
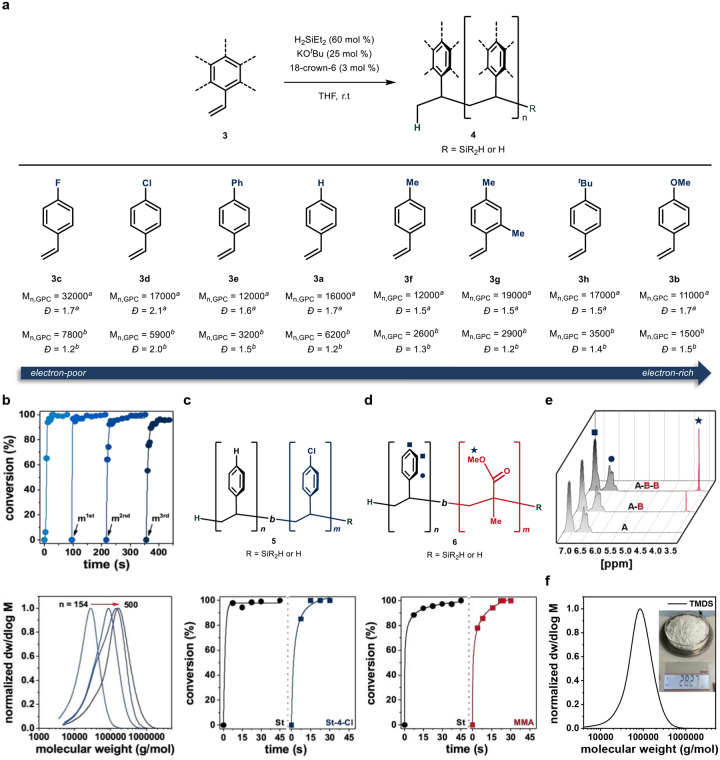
Scope and persistent characteristics of HAT-coupled SET polymerization. **a**, Scope of styrenes. Monomer conversions were >99% for all monomers except **3e** (>95%). Monomer conversion and M_n_ with *Đ* of polymers were monitored by real-time FT-NIR and GPC analyses. ^*a*^THF; ^*b*^m-xylene (reaction solvents). **b**, Homopolymerization of **3a**. Chain extension experiment showing conversion profiles and GPC traces upon three consecutive additions of **3a** at 100 s intervals (n: degree of polymerization). **c**, Block copolymerization of **3a** and **3d**: Conversion plots of poly(St-*b*-4-ClSt) (**5**). **d**, Block copolymerization of **3a** and methyl methacrylate (MMA): Conversion plots of poly(St-*b*-MMA) (**6**). **e**, Stacked ^1^H NMR spectra of block copolymers **6**, displaying chemical shifts from 3.3 to 7.3 ppm corresponding to **3a** (A) and MMA (B). **f**, Scale-up of HAP showing GPC traces obtained with TMDS. FT-NIR, Fourier transform-near-infrared spectroscopy; GPC, gel permeation chromatography; NMR, nuclear magnetic resonance spectroscopy.

To evaluate scalability and robustness, we examined HAP under aerobic conditions using unpurified styrene (St) derivatives containing commercial inhibitor additives. Efficient polymerization under these conditions underscores the operational simplicity and eliminates costly deoxygenation and drying procedures that often hinder the practical and industrial implementation of conventional radical and anionic polymerization methods^10,36,37^. First, we examined substituent effects across a range of electron-poor to electron-rich styrenes (**3a–3h**, Fig. 3a and Supplementary Fig. 3). Notably, the strongly electron-rich monomer **3b** polymerized efficiently, demonstrating the robustness of this radical initiation–anion propagation strategy. The resulting polymers showed comparable molecular weights (M_n_ = 12,000–32,000) and dispersity (*Đ* = 1.6–2.1)^28,38–40^. The tunability of HAP was demonstrated by simply changing the solvent to *m*-xylene, which afforded polymers with lower molecular weights and narrower dispersity. This M_n_ variation reflects solvent-dependent HAP efficiency, driven by altered aggregation state of alkali metal alkoxide with the HAT donor and the programmed M–π interactions in nonpolar, non-coordinating aromatic solvents such as xylene, vis-à-vis coordinating ethereal solvents (e.g., THF).

Achieving well-defined homopolymers and block copolymers is crucial for tailoring material properties in diverse applications such as coatings, packaging, and the automotive industry^41–43^. To test the feasibility of controlled homopolymerization, sequential additions of **3a** were performed at ca. 100 s intervals without introducing additional reagents (Fig. 3b). Each monomer addition resulted in rapid polymerization, with near-quantitative conversion of monomers. The degree of polymerization (n) increased steadily from 154 to 500, accompanied by linear increases in M_n_ (up to 52,000) and *Đ* (≈ 3.1) (Fig. 3b, Supplementary Fig. 4 and Supplementary Table 4). The relatively broad dispersity likely reflects heterogeneous chain growth, or non-uniform initiation efficiency during sequential monomer additions under aerobic conditions. These results demonstrate the persistent anionic character of HAP, facilitating efficient chain extension.

Next, chain extension experiments were conducted by introducing 4-chlorostyrene **3d** or methyl methacrylate (MMA) following the formation of polystyrene, with monomer conversion monitored by Fourier transform-near-infrared spectroscopy (FT-NIR) (Figs. 3c and 3d). GPC analysis revealed a modest increase in M_n_ {ca. 10,000 for poly(styrene-*b*-4-chlorostyrene) [poly(St-*b*-4-Cl-St), **5**] and 1,000 for poly(styrene-*b*-methyl methacrylate) [poly(St-*b*-MMA), **6**]} with *Đ* of 2.2 and 1.5, respectively (Supplementary Fig. 5 and Supplementary Table 5). Extending this strategy, sequential addition of MMA (**B**) following styrene **3a** (**A**) polymerization permitted the assembly of **A–B** and **A–B–B** architectures (>95% conversion). Successful incorporation of MMA was confirmed by ^1^H NMR spectroscopy (Fig. 3e, Supplementary Figs. 6–8 and Supplementary Table 6). The **B**/**A** (*n*:*m*) ratio in the copolymer, determined by ^1^H NMR integration (Supplementary information), increased systematically from 1:0.12 to 1:0.18, consistent with progressive incorporation of monomer **B**. This suggests that the persistency of the carbanions arises from effective charge dispersion by crown ether–bound alkali metals within an intimate ion pair cage, thereby underpinning the successful formation of both homopolymers and block copolymers.

Finally, the scalability of the HAP was evaluated in a fully open-air environment. Large-scale polymerization of **3a** using TMDS produced 28 g of PS with >99% conversion within 3 min (Fig. 3f). In addition, a block copolymer was obtained by introducing MMA, affording 13 g of poly(St-*b*-MMA) (M_n_ = 16,000 and *Đ* = 1.4) (Supplementary Table 7). Together, these results support the operational simplicity, robustness, and scalability of HAP for well-defined polymer synthesis under atmospheric conditions.

## Mechanistic Studies

**Fig. 4.**
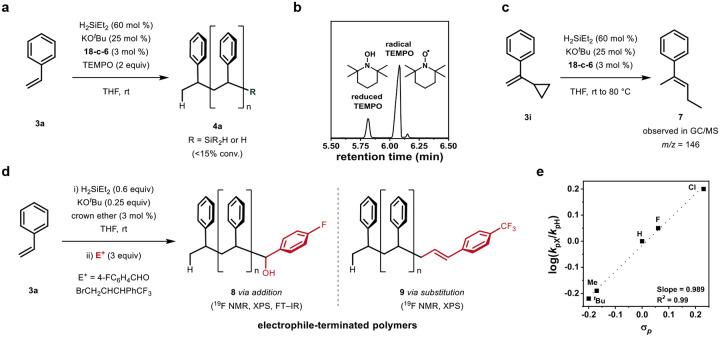
Mechanistic Studies. **a**, Radical trapping experiment using TEMPO. **b**, GC-MS analysis detecting the reduced TEMPO. **c**, Radical clock experiment. **d**, Electrophile trapping of carbanions within growing polymer chains. **e**, Hammett correlation analysis using *para*-substituted styrene.

To probe the radical-mediated nature of HAP, radical trapping experiments were conducted using TEMPO. In the presence of TEMPO, styrene polymerization was strongly suppressed (<15% conversion of **3a** to **4a**; Fig. 4a). Concurrently, gas chromatography–mass spectrometry (GC/MS) analysis revealed reduction of the TEMPO radical (Fig. 4b), confirming radical intermediacy. This result is consistent with a mechanism in which one-electron reduction of the benzylic radical to the corresponding benzylic anion occurs in a step distinct from HAT, providing a sufficient lifetime for interception by TEMPO^44^. The presence of radical intermediates was further validated by a radical clock experiment with **3i**, which produced the ring-opened product **7**, detected by GC/MS (Fig. 4c and Supplementary Fig. 9).

Next, to verify carbanionic propagation, protonation experiments were conducted by introducing excess MeOH during the course of the polymerization. Upon MeOH addition immediate cessation of polymer growth was observed, consistent with rapid protonation and quenching of the active anionic chain ends. Similarly, in chain-extension experiments, introduction of MeOH together with the second styrene monomer at the completion of the initial polymerization completely suppressed chain extension, and no further polymer growth was observed. To further probe the nature of the propagating chain ends, electrophile trapping experiments were performed by introducing *p*-fluorobenzaladehyde or *p*-trifluoromethylbenzyl bromide at the termination stage of polymerization (Fig. 4d). In both cases, formation of electrophile-terminated polymers (**8** and **9**) was confirmed by ^19^F nuclear magnetic resonance spectroscopy (NMR), Fourier transform-infrared spectroscopy (FT-IR), and X-ray photoelectron spectroscopy (XPS) analyses (Supplementary Figs. 10–15). Furthermore, Hammett analysis of *para*-substituted styrenes revealed a ρ value of 0.99, indicating a moderately polarized transition state (TS) accelerated by electron-withdrawing groups (EWGs), consistent with development of negative charge in the TS (Fig. 4e and Supplementary Table. 8). Together with our previous kinetic isotope effect (KIE) study identifying Si–H cleavage as the turnover-limiting step^20^, these results support a mechanism in which a transient radical intermediate–generated via turnover-limiting HAT from Si–H cleavage–is subsequently converted into a carbanion through SET. The interplay between radical persistency and single electron reduction thereby governs the overall dynamics of HAP.

**Fig. 5.**
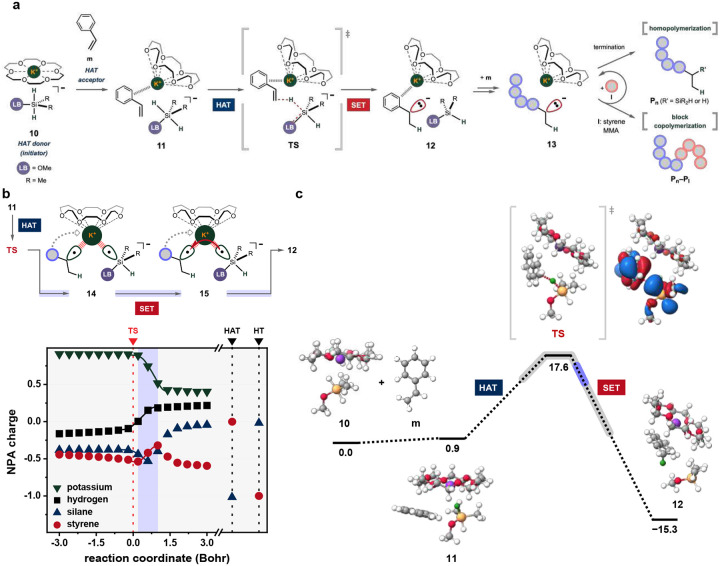
Proposed HCET Mechanism. **a**, Proposed mechanistic model for HCET polymerization. **b**, Proposed potassium-mediated SET mechanism (top). NPA charges for styrene along the intrinsic reaction coordinate (IRC) (bottom). All NPA charges were obtained from triplet single-point calculations at singlet IRC geometries; the TS corresponds to an IRC value of 0. Charges on styrene and silane fragments are compared for the hydrogen atom transfer (HAT) and hydride transfer (HT) pathways. **c**, Computed reaction energy profile for HCEP between styrene and a potassiated silane in the presence of **18-c-6**. Singly occupied molecular orbitals (SOMOs) of the reactant, TS, and product along the singlet pathway are shown.

Mechanistically, HAP proceeds through two distinct stages (Fig. 5a). In the initiation stage, Lewis base activation of hydrosilanes generates a crown ether–bound potassiated hypercoordinate hydridosilicon species **10**, functioning as the active HAT donor. Formation of a potassium salt bridge between **10** and styrene **m** within **11**, enables complexation-induced HAT to the β-carbon of **m** via a ternary transition state **TS**, generating transient complex **14** (Fig. 5b). This species comprises a carbon-centered radical and a four-coordinate open-shell silicon radical anion, held in close proximity by the alkali metal–crown ether bridge. The resulting diradical ternary complex subsequently undergoes cation-dependent SET to furnish ion-pair **12** via **15**. In this step, electron transfer proceeds from the putative four-coordinate, open-shell bisphenoidal silicon radical anion to the carbon-centered radical through the crown ether-bound potassium conduit [**K**^+^**(18-c-6)**]. This stepwise electron relay, governed by electrostatic interactions and reduction potential differences among the participating species (Supplementary Table 1), yields **12**. These carbanions are stabilized by noncovalent K^+^–π interaction, particularly with styrene units, and sustain anionic chain propagation. Upon monomer consumption, the resulting polymeric anions **13** undergo further chain growth, enabling both homopolymerization and block copolymerization with styrenes or MMA to afford **P**_**n**_ and **P**_**n**_–**P**_**I**_ copolymers, respectively. Overall, this mechanistic framework underscores the synergistic roles of alkali metal Lewis base activation of hydrosilanes, regioselective HAT, and potassium-mediated SET within a single ternary system, which together transform radical-driven initiation into controlled anionic propagation.

Density functional theory (DFT) calculations were performed to interrogate the HCET mechanism. The computed free energy profile (Fig. 5c) reveals the progression of HAT from silane to styrene, starting from the ternary complex and leading to the anionic product. The reaction begins with the silane in a bisphenoidal geometry, which is largely preserved at the TS, where substantial Si–H bond elongation occurs. The singly occupied molecular orbital (SOMO) of the TS shows pronounced electron density localized on the transferring hydrogen atom, consistent with a HAT process. This interpretation is further supported by the Natural Population Analysis (NPA) along the intrinsic reaction coordinate (IRC) (Fig. 5b). As the system approaches the TS, charge densities on both the silane and the transferring hydrogen atom remain nearly unchanged, indicating HAT without concurrent electron transfer. Immediately beyond the TS, the silane becomes more negatively charged, while both the hydrogen atom and the styrene fragment become less negatively charged, reflecting completion of HAT.

As the reaction progresses further along with the IRC, a reversal in charge distribution is observed (Fig. 5b and Supplementary Fig. 16). The silane returns toward a neutral charge state, while the positive charge on the potassium cation decreases to < 0.5 e, followed by a decrease in charge on the styrene fragment. This shift suggests a stepwise SET process in which the electron is transiently transferred from the silane radical anion to the potassium center and subsequently relayed to the styrene radical, producing the anionic styrene species. Consistent with this mechanistic scenario, binding energy analysis reveals that the **K**^+^**(18-c-6)** complex stabilizes the product state by approximately 3 kcal/mol relative to the reactant state (Supplementary Fig. 17) Calculated reduction potentials corroborate this sequence: the four-coordinate bisphenoidal silicon radical anion (*E*^*o*^ = −4.1 V) can thermodynamically reduce the crown ether-bound potassium cation (*E*^*o*^ = −3.4 V), which in turn reduces the styrene radical (*E*^*o*^ = −1.9 V) (Supplementary Table. 1). This potassium-mediated electron relay rationalizes the experimentally observed persistence of radical intermediates, as evidenced by TEMPO trapping (Fig. 4a), by temporally separating HAT from SET. Together, the DFT results support an HCET mechanism in which HAT-driven radical formation is followed by potassium-mediated one-electron reduction, generating anionic monomer **12** that sustains chain propagation.

## Conclusion

This work establishes a mechanistic foundation for hydrogen atom-coupled electron transfer (HCET) anionic polymerization (HAP) of styrene. This HCET strategy exhibits exceptional oxygen tolerance, employs environmentally benign reagents, and features operational simplicity with a broad monomer scope, providing access to well-defined homopolymers and block copolymers. Mechanistically, HAP is distinguished by coupling hydrogen atom transfer (HAT)-driven radical initiation with anionic propagation mediated by single electron transfer (SET) through crown ether–bound, Earth-abundant alkali-metal hypercoordinate silicon species. We uncover a SET pathway in which a four-coordinate, open-shell silicon radical anion transfers an electron to a carbon-centered radical via the crown ether–bound potassium conduit. By combining key features of radical and anionic polymerizations within a single process, this mechanism provides a conceptual and practical framework for developing more sustainable, versatile, and industrially relevant polymerization strategies. Even at this early stage, these findings expand the accessible chemical space of functional polymers and point toward new opportunities in advanced materials synthesis.

## Methods

### General

All monomers, reagents, and solvents from commercial sources were used as received without additional purification prior to polymerization. Please see Supplementary Methods for commercial sources of materials and characterizations as well as more detailed experimental and computational methods.

### Synthesis of polystyrene (PS)

The synthesis of polystyrene (PS) was performed by HAP method under aerobic conditions. Styrene (92 μL, 0.8 mmol), crown ether (0.02 mmol, 3 mol %), and THF (3 mL, 0.3 M) were added to a screw cap vial. H_2_SiEt_2_ (70 μL, 0.5 mmol, 60 mol %) was then added to the solution, and the reaction mixture was gently swirled at room temperature. Alkali metal Lewis base (25 mol %) was subsequently added to the solution. Upon completion of the reaction, the mixture was precipitated into cold methanol. This precipitation was repeated once or twice to further purify the polymer. The final polymer was dried in a vacuum oven. The conversions of the monomers were determined by real-time FT-NIR monitoring, and M_n_ and *Đ* were measured by using GPC.

### Homopolymerization

Styrene (92 μL, 0.8 mmol), 18-c-6 (4.9 mg, 0.02 mmol, 3 mol %), and THF (3 mL, 0.3 M) were introduced into a screw cap vial. H_2_SiEt_2_ (70 μL, 0.5 mmol, 60 mol %) was then added, and the mixture was gently swirled at room temperature to ensure homogeneity. KO^*t*^Bu (200 μL, 25 mol %) was introduced into the reaction mixture. After completion of the initial polymerization of styrene (1 min), additional styrene monomer (0.8 mmol per portion) was sequentially added at approximately 100 s intervals to continue the homopolymerization process. Upon completion of the reaction, the mixture was precipitated into cold methanol to isolate the polymer. The precipitation was repeated once or twice to further purify the polymer. The resulting polymer was dried in a vacuum oven to afford the final product. Polymer conversion was determined by real-time FT-NIR monitoring.

### Block Copolymerization

Styrene (92 L, 0.8 mmol), 18-c-6 (4.9 mg, 0.02 mmol, 3 mol %), and THF (3 mL, 0.3 M) were added into a screw cap vial. H_2_SiEt_2_ (70 μL, 0.5 mmol, 60 mol %) was added to the solution, and the mixture was gently swirled at room temperature. Next, KO^*t*^Bu (200 μL, 25 mol %) was introduced into the solution. After completion of the initial polymerization of styrene (1 min), a second monomer [0.8 mmol, 4-chlorostyrene (4-Cl-St) or methyl methacrylate (MMA)] was added sequentially to afford block copolymers poly(St-*b*-4-Cl-St) (**5**), and poly(St-*b*-MMA) (**6**), respectively. After completion of the reaction, the mixture was poured into cold methanol to precipitate the polymer. The precipitated polymer was collected, and the precipitation was repeated once or twice to enhance its purity. The purified polymer was then dried in a vacuum oven to obtain the final product.

#### Computational Methods.

All density functional theory (DFT) calculations were performed using the Gaussian 16 suite of programs^45^. Geometry optimizations of all stationary points, including transition states (TSs), were carried out at the uB3LYP-D3(BJ)^46–49^/6–31+G(d,p) level of theory (singlet spin state), using the polarizable continuum model (PCM) model^50^ with THF as the implicit solvent. All optimized geometries were validated by frequency analysis at the same level of theory and solvation model. Subsequently, single-point energy calculations were performed at the DLPNO-CCSD(T)^51–54^/def2-TZVP^55^ level of theory using the def2-TZVP/C auxiliary basis set, with THF as the implicit solvent. These DLPNO-CCSD(T) calculations were carried out using the ORCA 5.0 program^56^. Free energy values reported were based on the single point energies at the DLPNO-CCSD(T) level of theory with the thermal corrections computed at the uB3LYP-D3(BJ)/6–31+G(d,p) level.

## Supplementary Material

**Supplementary information** The online version contains supplementary material available at

Supplementary Files

This is a list of supplementary files associated with this preprint. Click to download.

- SIHAPmaster.pdf

## Figures and Tables

**Fig. 1.**
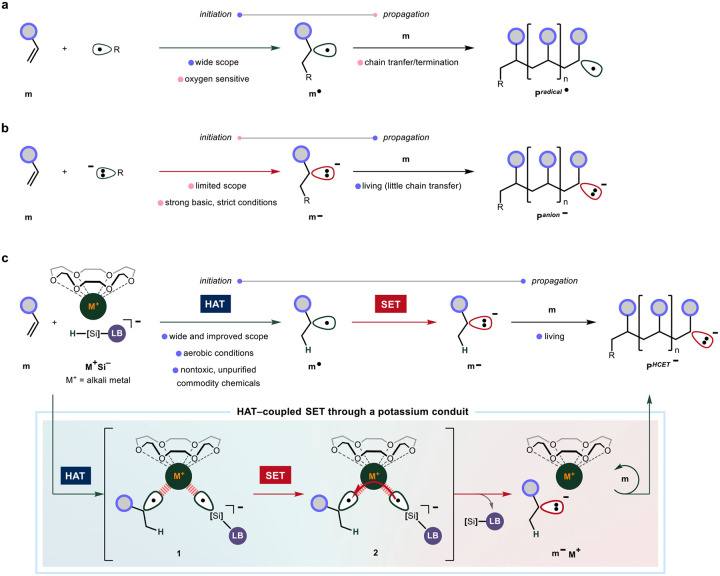
Olefin polymerization strategies involving changes in the oxidation state of the carbon center. **a**, General scheme and key features of radical polymerization. **b**, General scheme and key features of anionic polymerization. For simplicity, the metal cation is omitted. **c**, Illustration and key features of integrated, hydrogen atom–coupled electron transfer anionic polymerization (this work). The metal cation associated with the anionic species is omitted in the top panel for clarity, while the detailed ion pairing is shown in the bottom panel. Our mechanistic hypothesis is that this strategy proceeds through radical initiation, followed by anionic propagation. The SET step occurs via a potassium conduit, driven by electrostatic interactions between alkali metal cation and silicon radical anion. M, alkali metal; LB, Lewis base.

## Data Availability

All data supporting the findings of this study, including experimental details, spectroscopic characterization data for all compounds, and computational details, are available within the paper and its Supplementary Information, or from the corresponding author upon reasonable request.
